# African tropical rainforest net carbon dioxide fluxes in the twentieth century

**DOI:** 10.1098/rstb.2012.0376

**Published:** 2013-09-05

**Authors:** Joshua B. Fisher, Munish Sikka, Stephen Sitch, Philippe Ciais, Benjamin Poulter, David Galbraith, Jung-Eun Lee, Chris Huntingford, Nicolas Viovy, Ning Zeng, Anders Ahlström, Mark R. Lomas, Peter E. Levy, Christian Frankenberg, Sassan Saatchi, Yadvinder Malhi

**Affiliations:** 1 Jet Propulsion Laboratory, California Institute of Technology, 4800 Oak Grove Drive, Pasadena, CA 91109, USA; 2 Department of Geography, College of Life and Environmental Sciences, University of Exeter, Armory Building, Rennes Drive, Exeter EX4 4RJ, UK; 3 Laboratoire des Sciences du Climat et l'Environnement, Orme des Merisiers, bat. 701-Point courier 129, 91191 Gif Sur Yvette, France; 4 School of Geography, University of Leeds, Leeds LS2 9JT, UK; 5 Centre for Ecology and Hydrology, Benson Lane, Wallingford OX10 8BB, UK; 6 Department of Atmospheric and Oceanic Science, University of Maryland, 2417 Computer and Space Sciences Building, College Park, MD 20742-2425, USA; 7 Department of Physical Geography and Ecosystem Science, Lund University, Sölvegatan 12, 223 62 Lund, Sweden; 8 Centre for Terrestrial Carbon Dynamics, Department of Animal and Plant Sciences, University of Sheffield, Western Bank, Sheffield S10 2TN, UK; 9 Centre for Ecology and Hydrology, Midlothian, Penicuik EH26 0QB, UK; 10 Environmental Change Institute, School of Geography and the Environment, University of Oxford, South Parks Road, Oxford OX1 3QY, UK

**Keywords:** Africa, carbon, Congo, rainforest, tropic, uncertainty

## Abstract

The African humid tropical biome constitutes the second largest rainforest region, significantly impacts global carbon cycling and climate, and has undergone major changes in functioning owing to climate and land-use change over the past century. We assess changes and trends in CO_2_ fluxes from 1901 to 2010 using nine land surface models forced with common driving data, and depict the inter-model variability as the uncertainty in fluxes. The biome is estimated to be a natural (no disturbance) net carbon sink (−0.02 kg C m^−2^ yr^−1^ or −0.04 Pg C yr^−1^, *p* < 0.05) with increasing strength fourfold in the second half of the century. The models were in close agreement on net CO_2_ flux at the beginning of the century (*σ*_1901_ = 0.02 kg C m^−2^ yr^−1^), but diverged exponentially throughout the century (*σ*_2010_ = 0.03 kg C m^−2^ yr^−1^). The increasing uncertainty is due to differences in sensitivity to increasing atmospheric CO_2_, but not increasing water stress, despite a decrease in precipitation and increase in air temperature. However, the largest uncertainties were associated with the most extreme drought events of the century. These results highlight the need to constrain modelled CO_2_ fluxes with increasing atmospheric CO_2_ concentrations and extreme climatic events, as the uncertainties will only amplify in the next century.

## 1. Introduction

Covering an area of 2.3 million km^2^ (as defined by Zelazowski *et al.* [1]), the African humid tropical biome comprises 15% of global forests [2–5], yet dominates global inter-annual variability in terrestrial carbon cycling—about 50% of that from the global land mass, which is the most out of the pan-tropics, more than the entire Northern Hemisphere, and approximately as much as all of the oceans over the twentieth century [6–8]. African tropical rainforests strongly modulate regional climate, especially precipitation patterns, dominating global tropical rainfall during the transition seasons, and are tightly connected to global climate [9–13].

Despite its global and regional importance, this region has among the least environmental observations worldwide [6,13–17]. We have very few measurements on African humid tropical carbon stocks and fluxes [18,19], we do not know whether the biome is a net sink or source of atmospheric CO_2_, and we have little certainty as to the climate change response for the region [6,14]. Atmospheric CO_2_ flux inversions are subsequently among the most poorly constrained for this region [20,21].

The region is undergoing major change from land use and climate. Deforestation in Central Africa is accelerating, similar to deforestation patterns in the rest of the tropics [22,23]. The climate response, however, may be disproportionately extreme. The ‘great drought’ that began in the 1960s in the Sahel lasted well into the 1980s, with 20–40% less precipitation in the 30 years following 1960 relative to before [24–26]. The drought reached the northern Congo Basin, which appears to have continued to decline strongly in precipitation (at least in the short term), unlike the rest of the humid pan-tropics (which have showed little consistent trend apart from episodic droughts) [9,27–29]. Vegetation and soil carbon stocks and productivity have been shown to be highly correlated with annual rainfall in Africa [6]. Compounding this decrease in precipitation, there has been an overall warming trend in Africa at large [28,30,31]. Given less water and hotter temperatures, we may expect increasing water stress to decrease CO_2_ uptake, and move the biome towards a net source of CO_2_ to the atmosphere [27].

Paradoxically, models and measurements have shown an increase in vegetation productivity and biomass of 0.3–0.4 Pg C yr^−1^ in the African humid tropics since the 1960s [6,7,14,18,19,32–34] (compare with deforestation fluxes of 0.1–0.3 Pg yr^−1^ and fossil fuel emissions of 0.04 Pg yr^−1^) [35,36]. From these studies, water appears not to be the strongest limiting control on plant productivity in the African humid tropics; rather, plant productivity may be limited by radiation [32,37,38] or there may be no climatic constraints to productivity in this biome [39]. The recent increase in plant productivity has been attributed to the CO_2_ fertilization effect [7,14,18,19,28,40–45]. Still, free air CO_2_ enrichment (FACE) studies have shown that the CO_2_ enrichment effect can be curtailed by nutrient limitation [46–49]—a physical mechanism not included in most of these modelling studies [50]. The latest developments of nutrient limitation in global models have shown a major decrease in the ability of the terrestrial biosphere to sequester increasing atmospheric CO_2_ [51–58].

Given changes in atmospheric CO_2_ concentration, temperature and water throughout the twentieth century, we ask how have African humid tropical rainforests responded to these different climate forcings? Because of the sparseness of observation networks in the region for this period (apart from some plots from the AfriTRON network) [18], we rely on models. However, instead of relying on any one model, which may or may not represent an outlier to the community of models that encompass global uncertainty in climate change [42,59], we take advantage of the development of a new ‘network of models’, that is, a recent land surface model intercomparison project (MIP) called TRENDY (http://dgvm.ceh.ac.uk/node/21), which has united nine global land surface models through common forcing data over the period of 1901–2010 [60]. This extends a previous CarboAfrica Model InterComparison (CAMIC) project [37] that used four of these models over Africa for 1982–2006 (see also [61]). We are therefore able to add sophistication to our driving question, and ask how the net CO_2_ flux uncertainty—as defined by the model–model convergence/divergence—varies over this space and time domain.

## 2. Methods

The nine global land surface models from the TRENDY MIP include: CLM4-CN [55], HYLAND [62], LPJwsl [63], LPJ-GUESS [64], OCN [56], ORCHIDEE [65], SDGVM [66], TRIFFID [67] and VEGAS [68]. Model output for TRENDY was downloaded from: http://www-lscedods.cea.fr/invsat/RECCAP/. Output from multiple versions of the same model was sometimes available; in these cases, we used output only from the most recent version. We primarily used the version S2 runs, which correspond to simultaneously meteorological forcings and atmospheric CO_2_ concentration variation following twentieth century increases, with disturbance turned off and a constant land-use mask; this version represents the ‘natural’ state and change of the system. We also used version S1, which varies only CO_2_, to evaluate sensitivities to CO_2_ and climate.

The TRENDY models were driven primarily with CRU+ NCEP climate forcing data for 1901–2010 [60], downloaded at http://dods.extra.cea.fr/data/p529viov/cruncep/ through http://dgvm.ceh.ac.uk/node/9 [69–72]. The CRU+NCEP data are a combination of two existing datasets: (i) Climate Research Unit (CRU) TS.3.1 0.5°× 0.5° monthly climatology covering the period 1901–2009; and (ii) US National Oceanic and Atmospheric Administration (NOAA) National Centers for Environmental Prediction (NCEP) and National Center for Atmospheric Research (NCAR) reanalysis 2.5°× 2.5° 6-hourly climatology covering the period 1948–near real time.

**Figure 1.**
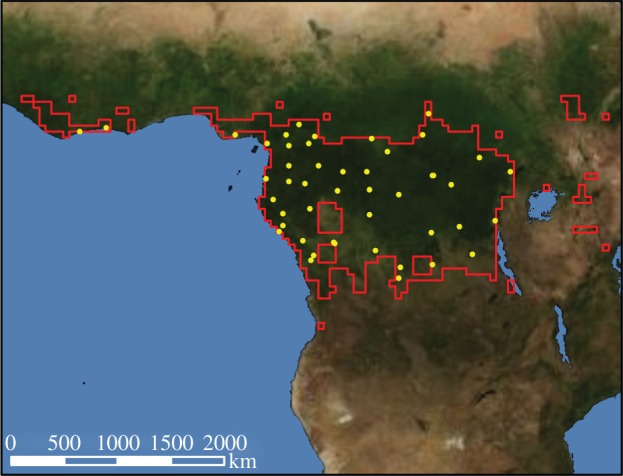
Map of African humid tropical forest delineation and station locations (*n* = 47) within mask used for climate forcing.

CRU TS.3.1 data were based on observed monthly mean temperatures and precipitation (among other variables) interpolated from more than 4000 weather stations distributed around the world. Station abundance in our study region was *n* = 47, though most were located closer to the coast rather than more inland into the Congo basin (figure 1). NCEP/NCAR reanalysis I data were created by assimilation into a climate model of meteorological observations from ships, satellites, ground stations, radar and the Rawinsonde observation programme. Input variables for the TRENDY models included: incoming longwave radiation, incoming shortwave radiation, total precipitation, air temperature, pressure, air-specific humidity, zonal wind (*u*) component and meridional wind (*v*) component. Incoming solar radiation was calculated from CRU-provided cloudiness, date and latitude; likewise, relative humidity was converted to specific humidity as a function of temperature and surface pressure (http://dods.extra.cea.fr/data/p529viov/readme.htm). For the CRU+NCEP merged dataset, climate data between 1948 and 2009 were based on CRU climatology, with NCEP used to generate the diurnal and daily variability. NCEP data were bi-linearly interpolated to the 0.5° × 0.5° CRU resolution for all fields except precipitation. Data for before 1948 and after 2009 were extrapolated from the CRU–NCEP statistical relationships for during 1948–2009. Atmospheric CO_2_ data were sourced from ice core + NOAA annual resolution for 1860–2010.

Model output variables assessed included: net biome production (NBP), gross primary production (GPP), heterotrophic respiration (Rh), autotrophic respiration (Ra), ecosystem respiration (Re) and net primary production (NPP). The models provided inconsistent and different combinations of variables, but enough variables so that those missing could be calculated. We calculated NPP for LPJ-GUESS from the available GPP minus Ra. We calculated Ra for HYLAND, ORCHIDEE and VEGAS from the available GPP minus NPP. We calculated Re from available Ra plus Rh (CLM4-CN, LPJ-GUESS, LPJ, OCN, SDGVM, TRIFFID) or NBP minus GPP (HYLAND, ORCHIDEE, VEGAS). HYLAND reported net ecosystem production (NEP), which we assume equal to NBP, though we note that technically NBP should include an additional flux from fire and other disturbance as well as lateral carbon transport that NEP would not include. HYLAND was reported in opposite sign for NEP so we reversed the sign. The models reported different time units (VEGAS in per years, LPJwsl in per months, all others in per seconds), so we converted all model results to per year.

We created a half-degree mask of the African humid tropics (figure 1; area = 2 312 749 km^2^) used to clip from the global model output, following Zelazowski *et al*. [1] for consistency with the previous analyses. We transformed the mask to match the relative varying native spatial resolutions of the models. We produced annual means for each of the variables by averaging the available 6-hourly model output and preserving the native spatial resolution for each model. For climate attribution, we selected the dominant driving variables that have undergone relatively large twentieth century shifts, i.e. precipitation and air temperature. We calculated trend correlations—that is, we did not remove trends to assess de-trended variability, as is often done—as we were more interested in what drives trends rather than inter-annual variability, though we additionally include a complementary analysis of inter-annual variability. We defined ‘uncertainty’ as inter-model standard deviation. We compared two time periods for pre- and post-1968, as defined as the start of the ‘great drought’ in the Sahel, which we test for extension into the humid tropics.

To assess the model sensitivity of the terrestrial carbon cycle, we follow the ‘feedback analysis’ approach of Friedlingstein *et al*. [42] for their uncoupled simulations. That is, the change in land carbon storage sensitivity to change in atmospheric CO_2_ concentration may be formulated as:2.1
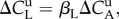
where 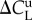 is the change in land carbon storage in the uncoupled simulation arising from an increase in atmospheric CO_2_ concentration of 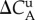, and *β*_L_ is the land carbon sensitivity to atmospheric CO_2_. Friedlingstein *et al*. use equation (2.1) to show the cumulative absolute change in land carbon storage from each of the uncoupled C^4^ MIP runs against atmospheric CO_2_ concentration for uncoupled simulations, which we also follow. To isolate the impact from ‘climate change’, Friedlingstein *et al*. give the following equation, which we adapt from their coupled runs:2.2
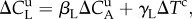
where 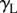 is the land carbon sensitivity to climate change with temperature increase of 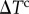. Subtracting equation (2.1) from equation (2.2):2.3
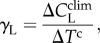
which can isolate the ‘climate alone’ impact on land carbon uptake. The resultant analysis shows the cumulative net CO_2_ flux over the twentieth century as the standard deviation between the models (e.g. uncertainty) forced with CO_2_ alone (e.g. TRENDY version S1), forced with varying CO_2_ + climate (e.g. TRENDY version S2), and the difference between the two, which is the impact of climate alone.

## 3. Results

**Figure 2.**
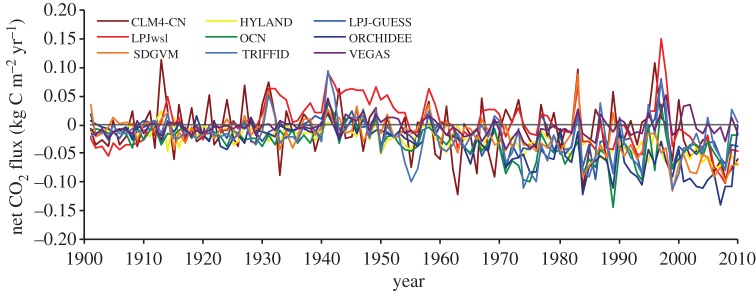
Mean annual African humid tropical net CO_2_ flux (negative is carbon sink) from nine dynamic global vegetation models in the TRENDY model intercomparison project.

**Figure 3.**
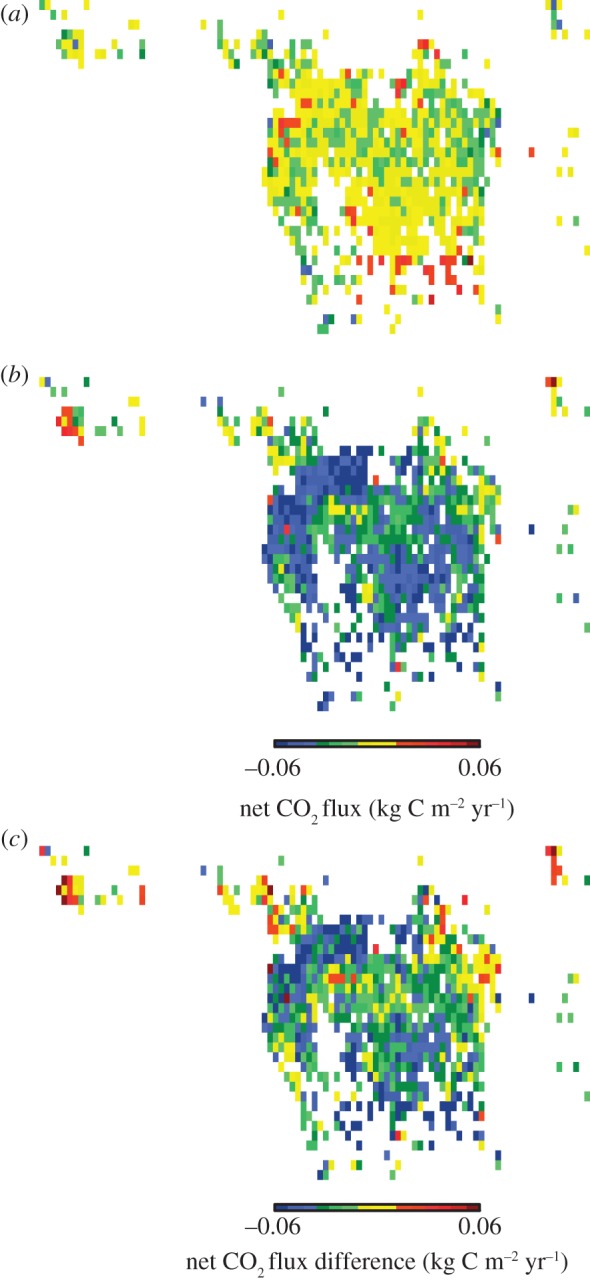
Multi-model (nine) mean net CO_2_ flux in the African humid tropics: (*a*) 1901–1967; (*b*) 1968–2010; (*c*) 1968–2010 minus 1901–1967.

The African humid tropics are estimated to be an overall significant natural (i.e. no disturbance) net carbon sink from 1901 to 2010, with a multi-model (nine) mean net CO_2_ flux of −0.018±0.009 kg C m^−2^ yr^−1^ or −0.04±0.02 Pg C yr^−1^ (*p* < 0.05; figure 2). The individual model largest carbon sinks came from ORCHIDEE, OCN and HYLAND (−0.03 kg C m^−2^ yr^−1^; table 1). LPJwsl was a non-significant overall carbon source (0.003 kg C m^−2^ yr^−1^). The multi-model sink strength increased by 4.3× post-1968 (pre-1968: −0.008 kg C m^−2^ yr^−1^; post-1968: −0.035 kg C m^−2^ yr^−1^). Apart from the edges, there was little spatial variability in the multi-model net CO_2_ flux for the biome (figure 3*a–c*). The component fluxes (GPP, NPP, Ra, Rh and Re) tended to display distinct stratification with little to no overlap among models (see the electronic supplementary material, figure S1). Overall, GPP was largest, followed by Re, then Ra, NPP and Rh.

**Table 1.** Decadal averaged net CO_2_ flux (kg C m^−2^ yr^−1^) per model and multi-model ensemble standard deviation for African humid tropics.

	CLM4-CN	HYLAND	LPJ-GUESS	LPJwsl	OCN	ORCHIDEE	SDGVM	TRIFFID	VEGAS	ensemble s.d.
1901–1910	−0.018	−0.010	−0.010	−0.036	−0.015	−0.006	−0.001	−0.013	−0.011	0.014
1911–1920	0.005	−0.011	−0.006	−0.002	−0.014	−0.007	−0.011	−0.011	−0.006	0.019
1921–1930	0.002	−0.015	−0.008	0.001	−0.024	−0.019	−0.007	−0.017	−0.005	0.018
1931–1940	−0.002	−0.017	−0.001	0.034	−0.021	−0.009	−0.005	−0.008	−0.003	0.023
1941–1950	0.003	−0.011	0.005	0.061	0.002	−0.005	0.000	0.012	0.010	0.027
1951–1960	−0.016	−0.021	−0.009	0.021	−0.025	−0.017	−0.006	−0.034	0.002	0.023
1961–1970	−0.038	−0.023	−0.026	0.001	−0.031	−0.027	−0.018	−0.017	−0.012	0.026
1971–1980	0.000	−0.031	−0.034	0.000	−0.063	−0.058	−0.031	−0.058	−0.021	0.029
1981–1990	−0.010	−0.040	−0.042	−0.014	−0.065	−0.063	−0.029	−0.036	−0.005	0.033
1991–2000	0.006	−0.049	−0.022	0.011	−0.046	−0.068	−0.050	−0.031	0.002	0.039
2001–2010	−0.055	−0.061	−0.044	−0.048	−0.043	−0.095	−0.073	−0.028	−0.001	0.032
*1901–2010*	*−0.011*	*−0.026*	*−0.018*	*0.003*	*−0.031*	*−0.034*	*−0.021*	*−0.022*	*−0.005*	*0.026*

**Figure 4.**
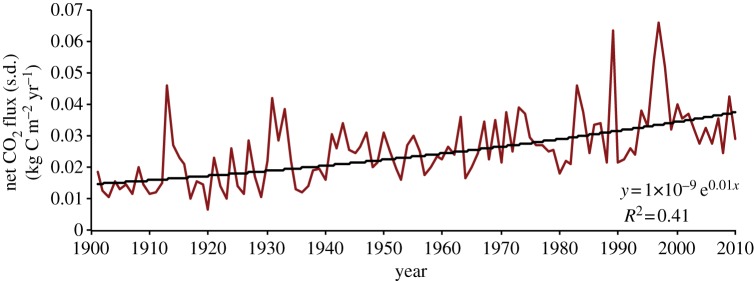
Multi-model (nine) annual standard deviation for African humid tropical net CO_2_ flux (red). An exponential curve (black) is fit through all years (equation in bottom-right).

The models were in relatively close agreement on the net CO_2_ flux in the beginning of the century and prior to the onset of the ‘great drought’ (*σ*_1901–1967_ = 0.021 kg C m^−2^ yr^−1^, increasing 0.00017 yr^−1^, *p* < 0.05; *σ*_1968–2010_ = 0.033 kg C m^−2^ yr^−1^, increasing 0.00020 yr^−1^, *p* < 0.05), but diverged from one another exponentially throughout the century (1 × 10^−9^e^0.01*x*^; *r*^2^ = 0.41, *p* < 0.05; figure 4). Uncertainty was 15% greater post-1968 relative to pre-1968, and 35% greater in 2010 than in 1901 (*σ*_1901_ = 0.019 kg C m^−2^ yr^−1^; *σ*_1910_ = 0.029 kg C m^−2^ yr^−1^).

**Figure 5.**
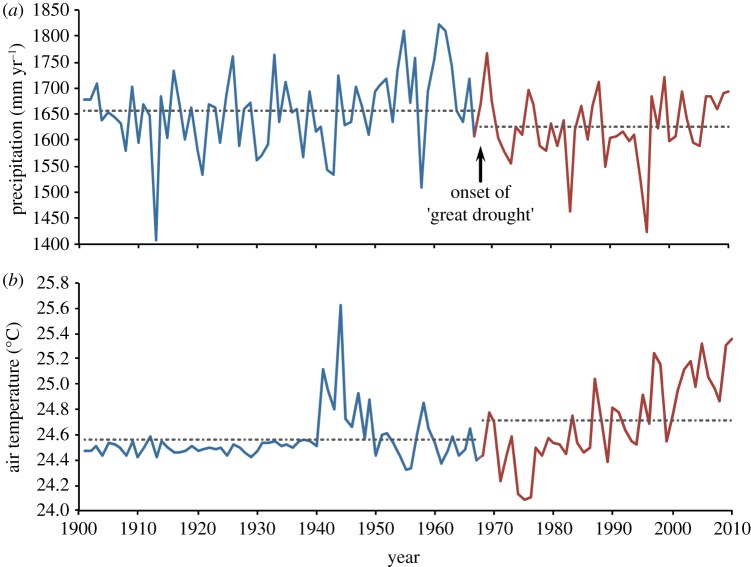
Mean annual climatological forcing for African humid tropics: (*a*) precipitation; (*b*) air temperature. Blue represents pre-1968, red represents post-1968. Dashed lines are the mean values through the 1901–1967 and 1968–2010 periods, respectively.

We show a significant (*p* < 0.05) decrease in precipitation (figure 5*a*) post-1968 in the African humid tropics. Mean precipitation from 1901 to 1967 was 1656 ± 9 mm yr^−1^, whereas mean precipitation from 1968 to 2010 was 1625 ± 10 mm yr^−1^. The post-1968 precipitation, while lower, was still relatively large—too large still to be considered a drought. Precipitation uncertainty near the equator globally is estimated to be approximately 30 mm [69], which is within a few % of the trend we observed, so had little effect on the statistical significance. While the overall spatially averaged biome showed a marginal decrease in precipitation, the explicit spatial patterns of precipitation change showed significant areas of precipitation decrease, particularly in the north; these were offset biome-wide by precipitation increases in the east and southwest (see the electronic supplementary material, figure S3*a–c*), similar to climate projections for the region [73].

Near surface air temperature was significantly (*p* < 0.05) hotter post-1968 in the region, though only marginally (figure 5*b*). Mean air temperature from 1901 to 1967 was 24.56 ± 0.02°C; from 1968 to 2010, mean air temperature was 24.71 ± 0.05°C, but increasing significantly (*p* < 0.05) at 0.02°C yr^−1^. Temperature uncertainty near the equator globally is estimated to be 1°C [69], which is within a few % of the trend we observed, so had little effect on the statistical significance. The temperature rise was greatest post-1980. The brief elevated temperature in the 1940s was due to one of the strongest El Niños of the century, which caused one of the highest anomalous air temperatures of the century in the Southern Hemisphere [74]. Air temperature primarily increased in the east and south of the biome, with little change in the northwest (see the electronic supplementary material, figure S3*d–f*).

We assessed which climatic and environmental factors influenced the model uncertainty. The differences between models are due to differences in sensitivities to climate forcings, rather than, for example, treatment of disturbance and land use, which were turned off in this analysis so as to focus on the natural ecosystem change to climate. The factor most correlated with the trend in net CO_2_ flux uncertainty was the trend in increasing atmospheric CO_2_ concentration (*r*^2^ = 0.31, *p* < 0.05). OCN, ORCHIDEE and HYLAND were the most sensitive to CO_2_, whereas VEGAS, CLM4-CN and LPJwsl were the least sensitive.

Second to CO_2_ was the trend in air temperature, which was weakly correlated with the trend in net CO_2_ flux uncertainty (*r*^2^ = 0.10, *p* < 0.05). This is primarily because the air temperature variation was relatively small; hence, the models were insensitive to air temperature in the region following the feed-back analysis (data not shown). The elevated temperature in the 1940s, for example, did not greatly impact the net CO_2_ flux uncertainty.

The trend in precipitation was not correlated with the trend in net CO_2_ flux uncertainty, though the two largest precipitation minima of the century (in 1913 and 1997), and to a lesser extent the minimum in 1983, corresponded with the largest increases in net CO_2_ flux uncertainty. This result cannot be understated given an expected increase in the number of extreme climate events in the twenty-first century [59].

**Figure 6.**
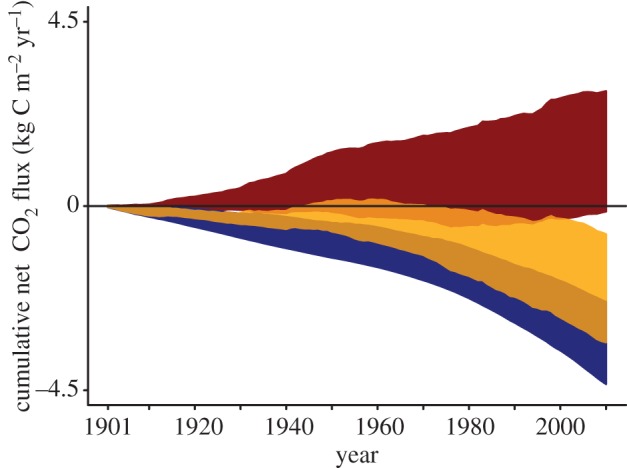
Sensitivity of land carbon storage to atmospheric CO_2_ (blue, sink), climate (red, source) and the combined effect of CO_2_ and climate (orange, sink) for African humid tropics over the twentieth century for TRENDY land surface models.

The change in climate alone moved the African humid tropical biome towards an increasing net carbon source, as temperatures increased and precipitation decreased, though also with increasing uncertainty (figure 6). However, the change in CO_2_ alone was a stronger driver of the biome towards an increasing net carbon sink, with moderately increasing uncertainty relative to that from climate. The uncertainty from climate alone at the beginning of the twenty-first century was 32% greater than that from CO_2_ alone. The strength of CO_2_ fertilization outweighed that from changing climate, so that the combined effect led to a net overall carbon sink.

**Figure 7.**
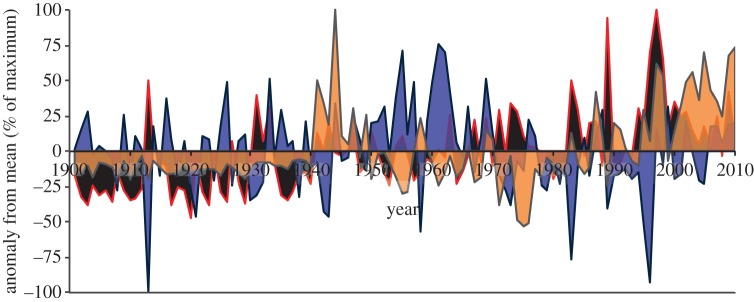
Inter-annual variability as defined as the annual anomaly from the long-term (1901–2010) mean, normalized to a percentage of the maximum value for: (i) the multi-model ensemble net CO_2_ flux standard deviation (black, red outline); (ii) precipitation (purple, blue outline); and, (iii) air temperature (orange, grey outline).

The inter-annual variability of air temperature, precipitation and net CO_2_ flux uncertainty illustrates the temporal influences of the magnitude changes of the climatic variables on the magnitude change of the net CO_2_ flux uncertainty (figure 7). It appears from figure 7 that there may be synergistic interactions between precipitation and air temperature, and their combined effect on net CO_2_ flux uncertainty, and a multiple regression integrating the two climatic variables yields a somewhat improved *r*^2^ of 0.17 (from 0.10 for air temperature alone). Including atmospheric CO_2_ concentration in the multiple regression increases the *r*^2^ to 0.35 (from 0.31 for atmospheric CO_2_ concentration alone). Integration of these drivers into an artificial neural network resulted in an equivalent *r*^2^ of 0.34, so no further structural explanation was found through these statistical approaches for the net CO_2_ flux uncertainty.

## 4. Discussion

Our analysis highlights three key results for the African humid tropics over the twentieth century in response to changing climate from nine land surface models: (i) the biome has been a natural net carbon sink, the strength of which increased over the century; (ii) uncertainty in the strength of the net carbon sink grew increasingly large throughout the century; and (iii) the trend in that uncertainty was largely related to increasing atmospheric CO_2_, not increasing water stress, though maxima were observed with the largest drought events.

As noted also in other modelling studies [7,14,17,37], as well as in an extensive ground survey by Lewis *et al*. [18], the influence of CO_2_ fertilization drove the biome towards an increasing net carbon sink. Lewis *et al*. measured an increase in aboveground carbon storage of 0.063 kg C m^−2^ yr^−1^ from 1968 to 2007, which is remarkably in the same order of magnitude as the multi-model carbon sink of 0.047 kg C m^−2^ yr^−1^ for the same period in this study, especially given entirely different estimation approaches, inexact spatial overlap and simplification of a very complex biome by the models. The models vary in how CO_2_ is allocated to wood, which has a long residence time and would show a greater biomass increase for the same unit increase in GPP relative to models that allocate NPP more towards pools with fast turnover times such as fine roots and leaves. Moreover, the uptake estimate of Lewis *et al*. is nearly identical to that from SDGVM (0.064 kg C m^−2^ yr^−1^). The multi-model mean C uptake is reduced primarily owing to the contribution from VEGAS, which estimated a net C loss, rather than gain, during that time period; removal of VEGAS from the multi-model mean changes the C uptake to 0.054 kg C m^−2^ yr^−1^.

Still, in general, the models estimated less C uptake than did Lewis *et al*., though with assumptions and biases in both the measurement and modelling approaches it is difficult to pinpoint the reasons behind the difference. Lewis *et al*. offer a number of hypotheses to explain their large C uptake, leaning towards changing resource availability and possibly species-specific advantages, though with nothing as of yet conclusive. It is possible that the models should be even more sensitive to CO_2_ fertilization than they already are, at least in this region, responding to the notion that in relatively species-rich areas some species may find CO_2_ fertilization particularly advantageous, and drive the whole biome towards a stronger carbon sink. Supporting this concept is observational evidence from Fauset *et al*. [29], who showed that biomass increased in Ghana from a shift in species composition, though the shift was related to the ‘great drought’ rather than CO_2_ fertilization.

Unlike exponentially rising atmospheric CO_2_, the CO_2_ fertilization effect on vegetation may not increase exponentially, instead decelerating with nutrient limitation [46,48,49,51–58]. In our study, most of the models do not have nutrient limitation mechanisms implemented; however, three do—SDGVM, CLM4-CN and OCN, the latter of which may be directly comparable with ORCHIDEE from which OCN is derived. Throughout the century, OCN generally tracked ORCHIDEE, which estimated the overall largest net carbon sink, but OCN departed significantly from ORCHIDEE in the past decade (*σ*_OCN-ORCHIDEE,1901–2000_ = 0.011 kg C m^−2^ yr^−1^; *σ*_OCN-ORCHIDEE,2001–2010_ = 0.039 kg C m^−2^ yr^−1^), aligning more similarly to CLM4-CN (*σ*_OCN-CLM4-CN,2001–2010_ = 0.017 kg C m^−2^ yr^−1^).

These patterns suggest that the CO_2_ fertilization effect may or has already begun to decrease in strength, though this would emerge more apparently as models start to integrate nutrient cycles. African rainforests may be moderately nutrient limited [47]. The difference in treatment of the CO_2_ fertilization effect is the primary reason for the increasing uncertainty in net CO_2_ flux throughout the twentieth century. Acclimation to high CO_2_ is not incorporated in most models, nor are there any *in situ* CO_2_ enrichment experiments in tropical forests with which to compare with models [75]. The tropical forest carbon sink has the largest uncertainty of all forests worldwide [34]. To help reduce the uncertainty, models need to integrate nutrient cycling, which would limit sensitivity to the CO_2_ fertilization effect particularly moving forward into the twenty-first century. Further assessment of *in situ* observations of C uptake is needed to constrain the magnitude and drivers of C uptake. Moreover, experimental data are needed to assess CO_2_ acclimation in the humid tropics, e.g. a tropical FACE experiment, as well as warming to confront models. Finally, the climatic forcing data for the region need to have better quantification of uncertainties, though presumably the climatic forcing data have increased in robustness through time, thereby tightening our confidence on the increasing model divergence.

Despite the strong decrease in precipitation post-1968 related to the ‘great drought’ in the Sahel, we found only a marginal decrease in precipitation overall for the African humid tropics (particularly for the northern part of the biome) [9] and little impact of water stress for our study. Certainly, parts of the biome were more impacted than others (see the electronic supplementary material, figure S3*a–f*), so averaging over a heterogeneously impacted area reduces the drought signal. Still, the reduction in precipitation and increase in temperature were sufficiently small enough not to produce a strong signal. Water stress would have manifested itself in GPP, because CO_2_ uptake should be controlled by stomatal conductance/resistance, which is controlled by water stress [76]. However, we found no relationship between GPP uncertainty and net CO_2_ flux uncertainty. In a recent study on ORCHIDEE in Africa, Traore *et al*. [17] found that GPP increased over the past 30 years even though soil moisture decreased, and that there was no relationship between precipitation and GPP variability in Central Africa (see also [16]), which supports our conclusions. Moreover, even with decreased precipitation, the African humid tropical region has persistent cloud cover and lower rates of evapotranspiration relative to, for example, Amazonia, so soil moisture remains at non-stress levels [77]. Further, if similar to Amazonia, then African humid tropical forests may also be deep rooted and able to withstand droughts [78].

Although there was a lack of response to the overall small drying trend in the humid African tropics, the greatest uncertainties resulted from direct response to the largest drought events throughout the twentieth century. The model divergence is linked to differences in soil moisture response thresholds across models. The potential decreasing CO_2_ fertilization strength may soon be outpaced by an increasing climate response (figure 6). With extreme climatic events expected to increase in the next century [59], it will be critical that improvement to land surface models focuses on representing the land response to these extremes accurately.
